# Quantitative and compositional responses of ammonia-oxidizing archaea and bacteria to long-term field fertilization

**DOI:** 10.1038/srep28981

**Published:** 2016-06-30

**Authors:** Chao Xue, Xu Zhang, Chen Zhu, Jun Zhao, Ping Zhu, Chang Peng, Ning Ling, Qirong Shen

**Affiliations:** 1 Jiangsu Collaborative Innovation Center for Solid Organic Waste Utilization and National Engineering Research Center for Organic-based Fertilizers, Department of Plant Nutrition, Nanjing Agricultural University, Nanjing, 210095, Jiangsu, China; 2 Agriculture Environment and Resources Center, Jilin Academy of Agricultural Sciences, Jilin 130033, China

## Abstract

Archaeal (AOA) and bacterial (AOB) ammonia-oxidizer responses to long-term field fertilization in a Mollisol soil were assessed through pyrosequencing of *amoA* genes. Long-term fertilization treatments including chemical fertilizer (NPK), NPK plus manure (NPKM), and no fertilization over 23 years altered soil properties resulting in significant shifts in AOA and AOB community composition and abundance. NPK exhibited a strong influence on AOA and AOB composition while the addition of manure neutralized the community change induced by NPK. NPK also led to significant soil acidification and enrichment of *Nitrosotalea*. Nitrosospira cluster 9 and 3c were the most abundant AOB populations with opposing responses to fertilization treatments. NPKM had the largest abundance of ammonia-oxidizers and highest potential nitrification activity (PNA), suggesting high N loss potential due to a doubling of nutrient input compared to NPK. PNA was strongly correlated to AOA and AOB community composition indicating that both were important in ammonium oxidization in this Mollisol soil. Total N and organic C were the most important factors driving shifts in AOA and AOB community composition. The AOA community was strongly correlated to the activities of all sugar hydrolysis associated soil enzymes and was more responsive to C and N input than AOB.

Worldwide, agricultural production has resulted in the disruption of soil nutrient cycling such that nutrient loss from these soils has accelerated. The goal of any agricultural production system is to optimize yield with minimal cost. This is normally accomplished by the use of nitrogen, phosphorus, and potassium fertilizers addition to both ameliorate these nutrient losses and increase crop yield. In terms of N losses, archaeal and bacterial ammonia-oxidizing microorganisms drive the rate-limiting step of nitrification and play an important role in soil N-cycling processes that result in the production of nitrite which is further oxidized to nitrate^1^,^2^. When nitrate is present in excess of plant uptake demand the potential for nitrate leaching and N_2_O emissions are increased, resulting in N loss^3^. Both ammonia oxidizing archaea (AOA) and bacteria (AOB) are ubiquitously distributed in terrestrial ecosystems^4^. Profiling of AOA and AOB communities can be achieved by targeted sequencing of their functional *amoA* genes and quantified using qPCR.

The effects of long-term fertilization on soil AOA and AOB community composition and abundance has been studied in a purple soil^2^, a calcareous fluvo-aquic soil^5^, a forest soil^6^, an upland red soil^7^, an acidic luvisols soil^8^, and a paddy soil^9^. *AmoA* harboring soil microbial community composition has been found to be largely influenced by soil pH, total nitrogen (TN) and soil organic matter (SOM). Long-term fertilization treatments significantly altered soil nutrient supply such that TN and SOM usually increased in manure treated soils while soil pH generally decreased in chemical fertilizer treatments^10^. However, the question as to how long-term fertilization influences soil properties and thus impacts soil AOA and AOB community composition, abundance, and activity in Mollisol soils is still unresolved. In addition, the ratio of AOA versus AOB abundances in soils may play an important role in impacting soil ammonium oxidation rates^11^. AOA has been reported to be more abundant in stressed environments such as low pH soils^7^,^12^,^13^,^14^, a purple soil^2^, and an alkaline sandy loamy soil^15^. However, the relative importance of these two groups in influencing nitrification rates in Mollisol soils remains unknown.

Long-term experiments involving various agronomic practices have been conducted at Gongzhuling Agro-ecological Experimental Station since 1990 for the purpose of studying how varying fertilization regimes influence soil characteristics and crop yield^16^,^17^. By utilizing this long-term study site, the overall purpose of this study was to evaluate the effect of long-term organic and chemical fertilization on the soil ammonium oxidizing microbial community. Three fertilization treatments were included: chemical fertilizer (NPK), NPK plus manure (NPKM), and no fertilization (CK). Soil AOA and AOB community composition and abundances were assessed using targeted sequencing and qPCR of the archaeal and bacterial *amoA* genes.

## Results

### Soil chemical properties and potential nitrification activity

After 23 years of fertilization, different agricultural management regimes altered the chemical properties of the soils (Table 1). Compared to the control (CK), chemical fertilizer application alone (NPK) significantly decreased soil pH (Tukey’s HSD, p < 0.05), while the additional application of organic amendment (NPKM) counteracted this acidification effect, resulting in pH values similar to the control (Tukey’s HSD, p > 0.05). Though no significant differences in available K were detected among the treatments (Tukey’s HSD, p > 0.05), higher fertilizer input resulted in higher soil available phosphorus (AP) with NPKM containing the highest AP followed by NPK and CK (Turkey’s HSD, p < 0.05). Application of NPK significantly decreased soil organic matter (SOM) compared to CK, while the additional organic amendment (NPKM) resulted in the highest SOM values (Tukey’s HSD, p < 0.05). In addition, NPKM exhibited the highest TN among all treatments (Tukey’s HSD, p < 0.05), while CK and NPK were similar. The control (CK) contained the highest C/N ratio followed by NPKM and NPK (Tukey’s HSD, p < 0.05).

Different agricultural management regimes also resulted in changes to the potential nitrification activity (PNA) (Table 1). Compared to the control (CK), chemical fertilizer application alone (NPK) significantly increased PNA by 260%, while the additional application of organic amendment (NPKM) further enhanced PNA by 610%.

### Soil enzyme activity

The activities of seven soil enzymes varied among different fertilization regimes (Table 2). Acid phosphomonoesterase activity varied significantly among treatments with the highest activity associated with NPK (Tukey’s HSD, p < 0.05). Likewise, *N*-acetyl-glucosaminidase was highest in NPK, with no significant difference between CK and NPKM. The other five soil enzymes, all associated with the hydrolysis of sugars, were consistently highest in NPKM (Tukey’s HSD, p < 0.05). In addition, β-cellobiosidase, sulfatase, and β-glucosidase were higher in NPK than CK (Tukey’s HSD, p < 0.05). An insignificantly higher rate of β-glucosidase was found in NPK than CK (Tukey’s HSD, p > 0.05). Lastly, α-glucosidase and β-D-xylosidase exhibited higher activity in CK than NPK (Tukey’s HSD, p < 0.05).

### Ammonia-oxidizing archaeal and bacterial community abundances

**Figure 1:**
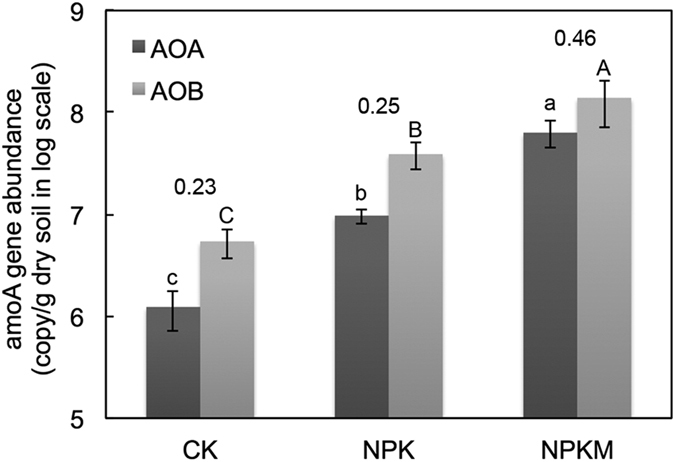
Abundances of ammonium oxidizing archaea (AOA) and bacteria (AOB). The AOA/AOB ratio of each treatment was shown on the top of the column.

Quantitative PCR was used to estimate the size of the ammonia-oxidizing archaeal and bacterial communities (Fig. 1). The abundance AOA in all treatments ranged from 1.2 × 10^6^ to 6.3 × 10^7^ copies g^−1^ dry soil, while that of AOB ranged from 5.4 × 10^6^ to 1.4 × 10^8^ copies g^−1^ dry soil. Application of NPK increased both AOA and AOB community population by 790% and 720%, respectively, compared to CK. In addition, manure plus fertilizer increased both AOA and AOB abundances by 650% and 360%, respectively, compared to NPK. NPKM had the highest AOA/AOB ratio (0.46) followed by NPK (0.25) and CK (0.23) (Fig. 1). Pearson correlation analysis revealed both AOA and AOB abundances were strongly correlated to soil TN (r = 0.94, p < 0.001 for AOA; r = 0.82, p < 0.001 for AOB) and PNA (r = 0.83, p < 0.001, for AOA; r = 0.70, p < 0.001, for AOB). No significant correlation was observed between soil pH and AOA/AOB abundances (r = 0.32, p > 0.05, for AOA; r = 0.26, p > 0.05, for AOB).

### Ammonia-oxidizing archaeal and bacterial community composition

**Figure 2:**
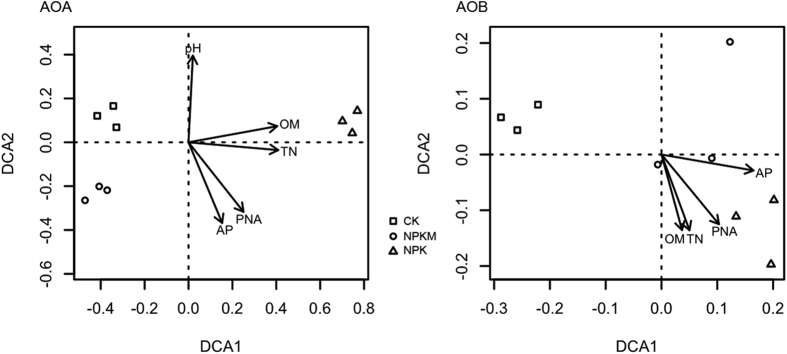
Detrended correspondence analysis (DCA) based on clusters to illustrate the beta-diversity (Bray-Curtis distances) between samples for AOA and AOB and to illustrate the correlations between community composition and environmental factors, including soil properties and potential nitrification activities (PNA). Only the environmental factors showing significant correlations were shown on DCA plot (p < 0.05).

**Figure 3:**
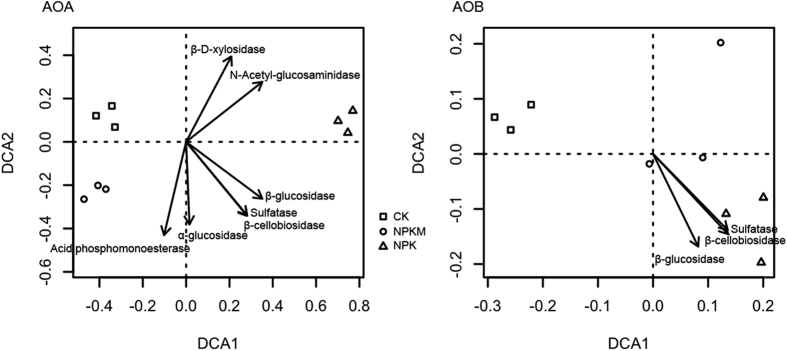
Detrended correspondence analysis (DCA) based on clusters to illustrate the beta-diversity (Bray-Curtis distances) between samples for AOA and AOB and to show the correlations between community composition and soil enzyme activities. Only the soil enzymes showing significant correlations were shown on DCA plot (p < 0.05).

The compositions of both the AOA and AOB communities varied significantly according to treatment (PERMANOVA: AOA, F = 72.209, p = 0.04; AOB, F = 6.309, p = 0.02) (Fig. 2). NPK harbored the most unique AOA and AOB community while CK and NPKM had relatively similar AOA community compositions. Using DCA, soil TN, SOM, AP, and PNA were correlated to both AOA and AOB community composition, while pH was only correlated to the composition of the AOA community (Fig. 2). Activities of all soil enzymes were closely correlated to AOA community composition, among which, β-glucosidase, β-cellobiosidase, and sulfatase were correlated to the composition of both AOA and AOB communities, while acid phosphomonoesterase, α-glucosidase, β-D-xylosidase, and N-Acetyl-glucosaminidase were only correlated to AOA community composition (Fig. 3).

**Figure 4:**
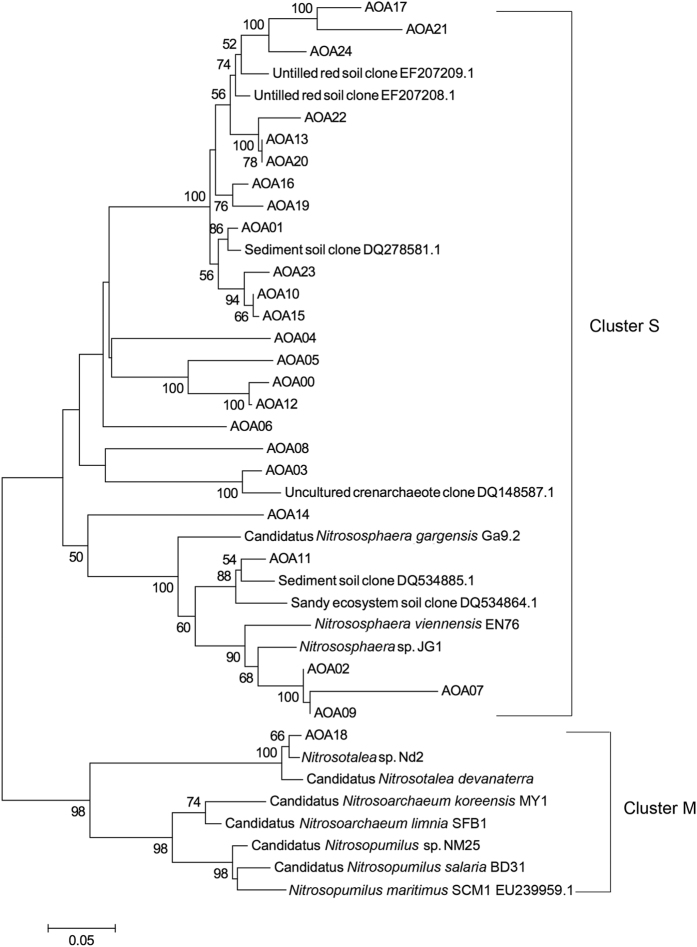
Neighbor-joining phylogenetic tree of the AOA OTU representative sequences of all under different fertilization treatments. Bootstrap values (>50%) are indicated at branch points. The scale bar represents 5% sequence divergence.

**Figure 5:**
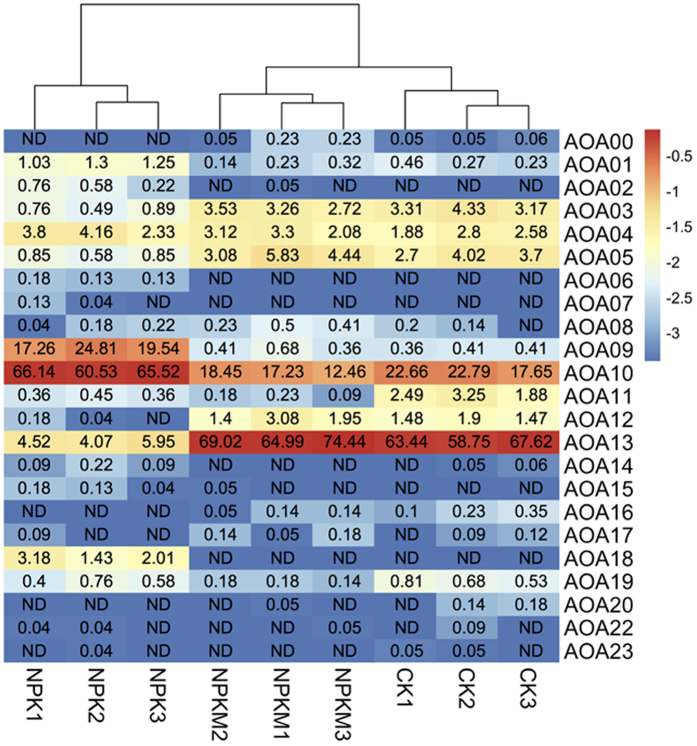
Heatmap showing the relative abundances of all AOA OTUs for all treatments. Colors from blue to red represent the least abundant to most abundant. Numbers in each cell represent the relative abundances of each OTU in the sample. ND = not detected.

Phylogenetically, all 25 AOA OTUs could be placed into three distinct groups (Fig. 4). Only OTU AOA18 was grouped into *Nitrosotalea*: OTUs AOA14, AOA11, AOA02, AOA07, and AOA09 were identified as *Nitrososphaera* spp. The other 19 OTUs were most related to uncultured environmental sample clones. The AOA community in all samples was dominated by two OTUs (AOA10 and AOA13), which were identified as uncultured soil clones and accounted for 33.7% and 45.9% of the total reads, respectively (Fig. 5). In addition, OTUs AOA10, AOA13, OTUs AOA01, AOA03, AOA04, AOA05, AOA09, AOA11 and AOA19 were also the commonly detected in all samples. OTU AOA09 (*Nitrososphaera* sp.) was also in very high abundance in the NPK treatment, and accounted for 20.5% of the total reads. NPK application led to an enrichment of OTUs AOA09 and AOA10 and a significant decrease in the relative abundance of OTU AOA13 (Tukey’s HSD, p < 0.05). OTUs AOA06 (*Nitrososphaera* sp.) and AOA18 (*Nitrosotalea* sp.) were identified as unique OTUs in the NPK treatment as they were not detected in any CK and NPKM samples (Fig. 5). AOA00 and AOA16 were detected in both NPKM and CK treatments, but were both absent in the NPK treatment (Fig. 5).

**Figure 6:**
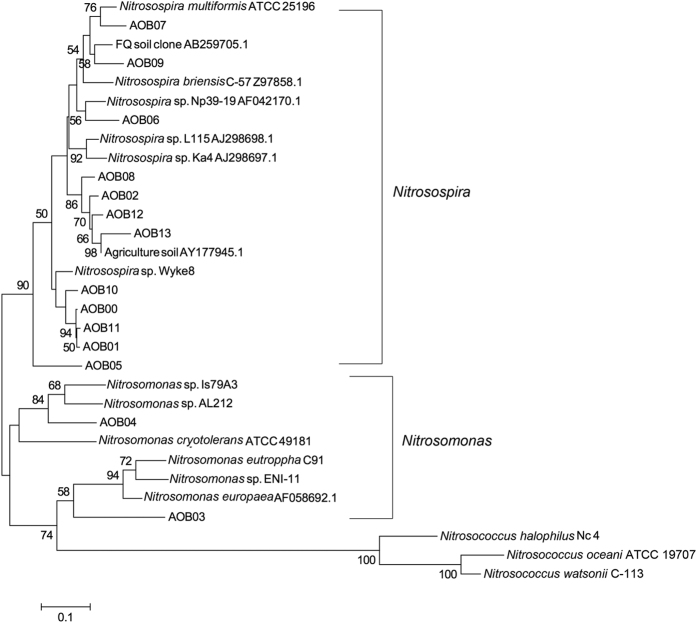
Neighbor-joining phylogenetic tree of the representative sequences of all AOB OTUs under each fertilization treatment. Bootstrap values (>50%) are indicated at branch points. The scale bar represents 10% sequence divergence.

**Figure 7:**
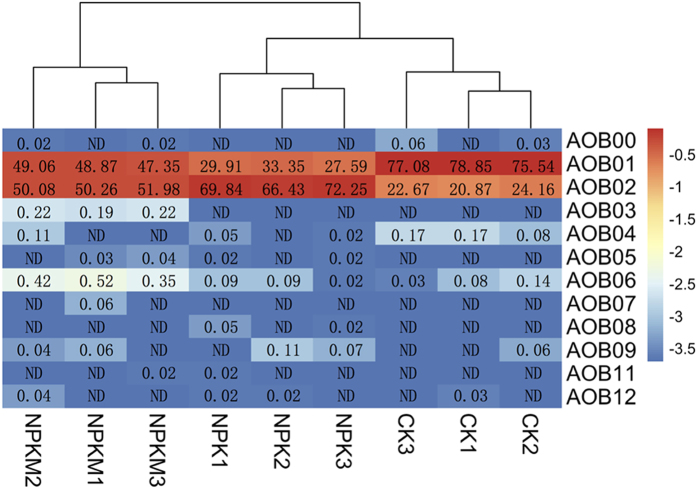
Heatmap displaying the relative abundances of all the AOB OTUs for all treatments. Colors, from blue to red, represent the least abundant to most abundant. Numbers in each cell represent the relative abundances of each OTU in the sample. ND = not detected.

Based on the phylogenetic tree, all the 14 AOB OTUs could be separated into two main branches with 7 clusters (Fig. 6). OTUs AOB04 (cluster 8) and AOB03 (cluster 7) were identified as *Nitrosomonas* spp., while the other 12 OTUs were most related to *Nitrosospira* spp. The AOB community of all samples was dominated by two OTUs, AOB01 (cluster 3c) and AOB02 (cluster 9), related most closely to *Nitrosospira* spp., and accounted for 52% and 47.6% of the total reads, respectively (Fig. 7). In addition, CK harbored the highest relative abundance of OTU AOB01 (77.2% of total reads) (Tukey’s HSD, p < 0.05). The application of NPK significantly decreased the abundance of OTU AOB01 to 30.3%, while the addition of manure (NPKM) significantly increased it’s relative abundance to 48.4%. However, it was still significantly lower than CK (77.2%) (Tukey’s HSD, p < 0.05) (Fig. 7). The response of OTU AOB02 was opposite to that of OTU AOB01 with the highest abundance in NPK (69.5%) followed by NPKM (50.8%) and CK (22.6%) (Tukey’s HSD, p < 0.05) (Fig. 7). OTU AOB06 (*Nitrosospira* sp.) was also shared among all samples with the highest relative abundance in NPKM (0.43%). Treatment NPKM had one unique OTU (AOB03 accounting for 0.21% of the reads) identified as *Nitrosomonas* sp., not detected in any other sample (Figs 6 and 7).

## Discussion

In this study, ammonia oxidizer communities in Mollisol soils after 23 years of different field fertilization treatments were investigated by qPCR and targeted sequencing of the *amoA* gene. We found that long-term field fertilization led to alterations in soil characteristics that influenced ammonia oxidizer community composition, abundance, nitrification activity and soil enzyme activities in a Mollisol soil. Application of chemical fertilizer alone decreased soil organic matter (SOM) and the C/N ratio, but increased soil available P (AP) with no influence on soil total N (TN). These results indicate that the NPK treatment was likely a more efficient treatment in that soil TN was the same as the control treatment, but resulted in a higher maize yield. However, the NPK treatment also decreased SOM due to little organic matter input with annual maize harvest, resulting in a smaller C/N ratio. This suggests that intensive cropping with only a chemical fertilizer input will result in SOM degradation. This loss of SOM cannot likely be ameliorated by rhizodeposition of C4 plant photosynthetic products. In addition, long-term field NPK treatment also led to soil acidification, as described in other studies^18^,^19^,^20^. The decreased pH resulted in high acid phosphomonoesterase activity, which further explained the higher AP in the NPK treatment. Since the optimal pH for *N*-acetyl-glucosaminidase is around 5.0, the decrease in pH from 7.53 to 5.96 predictably increased the activity of N-acetyl-glucosaminidase^21^. NPK application also stimulated the activities of cellulose degrading but decreased lignin degrading associated enzymes. These trends are, supported by a previous study on grassland and forest sites by Keeler *et al*.^22^. The addition of organic manure, which resulted in increases in soil TN, SOM and AP, also neutralized the acidification effect caused by chemical fertilizer application alone, indicating that the organic addition was able to buffer soil pH and sustain soil productivity and quality^23^. The additional manure application also increased the activities of all sugar hydrolysis associated enzymes detected in this study and was likely due to the stimulation of degradative soil microbial populations and increases in the abundances of manure-based enzyme substrates^24^.

Both AOA and AOB community composition varied according to long-term field fertilizer treatments, which agreed with previous studies on a purple soil^2^, a calcareous fluvo-aquic soil^5^, a forest soil^6^, an upland red soil^7^, an acidic luvisols soil^8^, and a paddy soil^9^. Alterations in soil chemical properties due to fertilizer treatment appeared to principally influence this shift. Of these, the significant correlation of TN, SOM, and AP to AOA and AOB community composition was specifically identified in a purple soil^2^. Overall, soil TN was the top contributor to the variations in AOA and AOB community composition. Nitrogen was applied as urea in the NPK and NPKM treatments and was likely rapidly hydrolysed, resulting in a high ammonium concentration that directly triggered shifts in community composition^25^. However, the additional application of organic manure (NPKM) appeared to mitigate the negative effects of the chemical fertilizer, resulting in AOA/AOB communities similar to the control. There is also evidence that *amoA* harboring soil microbial community composition is largely driven by soil pH. However, we found that soil pH was only correlated to AOA community composition, in agreement with a previous study focusing on acidic soil^12^, but inconsistent with another study on alkaline sandy loam soil^26^. The lack of significant correlation between pH and AOA/AOB abundances indicated that the impact of pH was constrained as a selective pressure on community composition only.

Potential nitrification activity (PNA) increased with N addition accordingly and was strongly correlated to both AOA and AOB abundances, indicating that these increases (CK < NPK < NPKM) likely contributed to higher potential nitrification. The strong correlation of PNA to changes in both AOA and AOB community composition indicated a contribution of an additional underlying functional shift, especially for the NPKM treatment (Fig. 2). Within soil, previous studies have found only AOB correlations with PNA, suggesting an important role in ammonium oxidizing capacity^5^,^6^,^27^. Studies that identified AOA as the dominate ammonium oxidizing community generally associated with relatively extreme environments such as red soil^13^ and marine systems^28^. Climatic conditions may play an important role in determining the influence of AOA versus AOB communities. The optimal temperature for most AOB is around 30 °C, while AOA are present in across a broad temperature range, from 4 °C in the deep ocean^29^ to 95 °C in a hot spring^30^. In this study, the experiment site has a mean annual temperature of 4–5 °C. This lower temperature may increase the importance of AOA regulated ammonium oxidization in these Chinese Mollisol soils. As such, both AOA and AOB communities appear important, though their actual individual contributions to ammonium oxidation remains unknown.

It has been established that ammonium oxidizers are mainly autotrophic^31^, however whether they are heterotrophic or mixotrophic is still in question. Tourna *et al*. reported that although *Nitrososphaera viennensis* is able to grow chemolithoautotrophically, considerable growth rates are obtained only upon the addition of low amounts of pyruvate^32^. *Nitrosomonas europaea* was considered an obligate chemolithoautotroph, later it was found to utilize fructose as a C source^33^. Jia and Conrad, using a stable isotope labeling technique, demonstrated that AOA were able to grow without consuming CO_2_^34^. Our data shows that both AOA and AOB were stimulated by the NPKM treatment and that AOA was more responsive to this organic input. In addition, the AOA community was strongly correlated to all enzymes involved in sugar hydrolysis while the AOB community was only correlated to two sugar hydrolysis associated enzymes (Fig. 3). These data suggests that, in these soils, AOA and AOB may exhibit mixotrophic or heterotrophic lifestyles that contribute to their ammonia oxidizing capacity. Moreover, comparing AOB to AOA responses, though both the AOA and AOB communities were influenced by chemical fertilizer addition alone (NPK), the “neutralization” effect by manure amendment was stronger on the AOA community composition, as the AOA community composition became more similar to the control than AOB (Fig. 2). Therefore, AOA mixotrophic/heterotrophic metabolism may be more prevalent in AOA than AOB.

In this study, regardless of treatment, AOB was always more abundant than AOA. This finding is contrary to most studies on acidic soils^7^,^12^,^13^,^14^, a purple soil^2^, and an alkaline sandy loamy soil^15^, where AOA was more dominant than AOB. However, AOB dominance has been identified in some studies, such as an agricultural soil in Germany^34^ and a grassland soil in New Zealand^35^. This is likely due to a combination of contrasting whole bacterial community and functional community compositions that vary due to geographic, climatic, and vegetation impacts, as well as land use histories (among others), which serve to shape the microbial community structure and function over time^36^,^37^. As such, these contrasting microbial communities, due to varying composition, would be expected to respond differently to external stressors. For example, Nicol *et al*. reported that AOA abundance increased with decreasing pH^14^, while in this study, pH had no obvious influence on the population of AOA and AOB but was instead more responsive to C and N inputs.

All the AOA OTUs were classified into three groups: the uncultured group and *Nitrosospheara* group, with only one OTU AOA18 identified as *Nitrosotalea* sp. This is in agreement with previous studies on alkaline sandy loam^26^, sandy loam soil^38^, and upland red soil^7^. Due to the general lack of AOA pure cultures, a large proportion of AOA *amoA* sequences in the database have been obtained from environmental samples. Thus 76% of the AOA OTUs could not be classified in this study. In addition, OTU AOA18 was the unique OTU for the NPK treatment, which was further identified as *Nitrosotalea* sp., of which *Nitrosotalea devanaterra* is the only cultivated obligatory acidophilic AOA species to date^39^. NPK treatment caused soil acidification resulting in an enrichment of *Nitrosotalea* sp., demonstrating that pH contributed to variations in the AOA community composition. Thus, *Nitrosotalea* sp. may be a candidate for use as an indicator for soil acidification in these Mollisol soils.

All the AOB OTUs were classified as *Nitrosospira* spp. and *Nitrosomonas* spp., in agreement with previous studies on alkaline sandy loam^26^, sandy loam soil^38^, and upland red soils^7^. OTU AOB03 (*Nitrosomonas* sp.) was a unique OTU, present only in NPKM treatment and may be associated with the metabolic use of organic compounds^33^. Overall, the AOB community was dominated by OTUs AOB01 and AOB02. These two OTUs exhibited opposite responses to fertilization treatments in that NPK enriched AOB02 (cluster 9), but decreased AOB01 (cluster 3c). Cluster 3c is a common AOB cluster widely distributed in soils at most ammonium concentrations and temperatures^40^. High abundances of cluster 9 OTUs has been rarely detected in agriculture soil. For example, Avrahami *et al*. found that *Nitrosospira* cluster 9 could only be detected at low ammonium concentrations, inconsistent with our results^40^. *Nitrosospira* cluster 1 has been found to be dominant at low temperatures (4~10 °C), but were absent after extended incubations at a low fertilizer treatment^40^. In this study, *Nitrosospira* cluster 1 was not detected in any soil sample. Together, these results indicate that different AOB strains, though phylogenetically grouped in the same cluster, may exhibit different adaptations to soil environment conditions.

## Conclusions

Long-term fertilization treatments including chemical fertilizer (NPK), NPK plus manure (NPKM) and no fertilization (CK) over 23 years altered soil properties resulting in significant shifts in the ammonium oxidizing archaeal and bacterial community and soil enzyme activities. NPK exhibited a strong influence on AOA and AOB community structure, while the addition of manure neutralized the community change as observed in the treatment with chemical fertilizer alone. NPK also led to significant soil acidification and enrichment of *Nitrosotalea* sp., which may be used as an indicator for soil acidification. *Nitrosospira* cluster 9 and 3c were the most abundant AOB groups and exhibited opposite responses to fertilization treatments. Populations of AOA and AOB were significantly correlated to soil total N and potential nitrification activity. NPKM had largest population of ammonium oxidizing community and highest potential nitrification activity, suggesting high N loss potential due to higher nutrient input, compared to NPK. PNA was closely strongly correlated to both AOA and AOB communities indicating both AOA and AOB were important in soil ammonium oxidizing in Mollisol. Lastly, the strong correlation of AOA to sugar hydrolysis enzyme activities indicated that AOA was more responsive to organic C and N inputs than AOB in these soils.

## Material and Methods

### Field experiment design

The long-term field fertilization experiment, initiated in 1990, is located at the Gongzhuling Agro-ecological Experimental Station (124°48′33.9′′E, 43°30′23′′N) in the Northeast China Plain, a representative agricultural area in Jilin Province, China. It has a mean annual temperature of 4–5 °C and 450–600 mm of mean annual precipitation. The cropping regime is dominated by one maize crop per year^41^. The soil was classified as a Mollisol developed from Quaternary loess-like sediments. The original soil composition is as follows: Organic matter 22.8 g/kg, total N 1.40 g/kg, total P (P_2_O_5_) 1.39 g/kg, total K (K_2_O) 22.1 g/kg, available P (P_2_O_5_) 27 mg/kg, and available K (K_2_O) 190 mg/kg and the soil pH was 7.6.

Three treatments with three replicates each were included, as described in a previous study (400 m^2^ for each replicate)^10^: (i) Field treated with chemical fertilizer containing N, P, and K (NPK), (ii) Field treated with NPK plus organic manure (NPKM), and (iii) Field without any fertilization serving as a control (CK). Chemical fertilizer was applied as annual rate of 165 kg N ha^−1^, 82.5 kg P_2_O_5_ ha^−1^, and 82.5 kg K_2_O ha^−1^. The N, P, and K were applied as urea, superphosphate, and potassium chloride, respectively. In the NPKM treatment, the same rates of chemical fertilizers as NPK treatment were used in addition to 60,000 kg ha^−1^ of organic pig manure containing 10–15% organic matter, 0.15–0.55% TN, 0.1–0.5% P_2_O_5_, 0.35–0.45% K_2_O, and 35–40% water content.

### Soil sampling and DNA extraction

Soil samples with three replicates from each treatment were collected before planting maize in May 2013. Ten soil cores (5 cm diameter) were collected at a depth of 0–20 cm from each plot. All samples were carefully mixed to form a composite sample. Moist soils were gently broken apart along the natural break points and passed through a 2-mm sieve to remove visible organic debris. After thorough mixing, the total DNA of each sample was extracted using an Ultra Clean™ Soil DNA Isolation Kit (MOBIO Laboratories, Carlsbad, CA, USA) according to the manufacturer’s instructions.

### Amplification and 454 pyrosequencing

Primer set amoA-1F (adapter-mid- GGGGTTTCTACTGGTGGT) and amoA-2R (adapter-mid- CCCCTCGGGAAAGCCTTCTTC) was used to amplify the *amoA* genes of bacteria and amoA-F (adapter-mid-STAATGGTCTGGCTTAGACG) and amoA-R (adapter-mid-GCGGCCATCCATCTGTATGT) were used to amplify the *amoA* genes of archaea, according to previously published protocols^42^,^43^, where “mid” refers to a unique barcode sequence used for sample sorting. Amplicons were sequenced on the 454 Life Sciences Titanium platform by Bion Biotech Co., Ltd. (Nanjing, China).

### Real-time PCR assay

To generate external standard curves for real-time PCR assay, the *amoA* genes of archaea and bacteria were amplified from soil DNA with each pair of primers listed above without adapter and mid, respectively. The PCR products were gel-purified and cloned to *Escherichia coli* using the pMD19-T cloning kit (Takara). After blue-white screening, the selected clones were sequenced and stored at −80 °C for future use. Plasmid DNA was extracted from the positive clone with the AxyPrep Plasmid Miniprep Kit (Axygen Bio, USA). Plasmid DNA concentrations were determined on a NanoDrop ND-2000 spectrophotometer (NanoDrop, Wilmington, DE, USA) and the copy numbers of each target gene was calculated. Ten-fold serial dilutions ranging from 1 × 10^2^ to 1 × 10^8^ copies of a known copy number of the plasmid DNA were subjected to real-time PCR assay in triplicate to generate an external calibration curve. Amplification efficiencies of 94.8~95.2% were obtained with R^2^ values of 0.999.

Real-time PCR was performed using biological triplicates and three technical replicates with two negative controls on an ABI 7500 Cycle (Applied Biosystems, Germany) under the following thermocycling conditions: 30 s at 95 °C, followed by 40 cycles of 5 s at 95 °C and 34 s at 60 °C. The reaction mixture contained 10 μl of the Premix Ex TaqTM (2×) (Takara), 0.4 μl of ROX Reference Dye II (50×), 0.2 mM of each primer, and 2 μl of template DNA with a final volume of 20 μl. Amplification specificity was verified by melting-curve analysis and agarose gel electrophoresis. Copy numbers were log10-transformed to normalize the values prior to statistical analysis.

### Soil analysis

Soil organic matter (SOM), pH, total nitrogen (TN), available phosphorus (AP), and available potassium (AK) was measured as described in previous study^44^. Soil pH was determined with a compound electrode (PE-10, Sartorious, Germany) in a 1:2.5 soil/water ratio solution. SOM was determined by dichromate oxidation, and TN was measured by a vario MACRO cube element analyzer (Elementar Analysensysteme, Germany). The AP was extracted by sodium bicarbonate and then determined following the molybdenum-blue method while AK was measured using a flame atomic absorption spectrophotometer.

### Assay of potential nitrification activity

Potential nitrification activity was measured as described previously^45^. Briefly, fresh soil samples (15 g) were placed in Erlenmeyer flasks with 100 ml of a 1.5 mM NH_4_^+^ and 1 mM PO_4_^3−^ mixture with the pH adjusted to 7.2. The slurry was then shaken on an orbital shaker at 180 rpm for 24 h at 25 °C to maintain aeration in the dark. Aliquots of 5 ml were subsequently removed using a wide-mouth pipette at 2, 6, 12, 22 and 24 h after the start of the incubation. The aliquots were then centrifuged, and the supernatant was filtered and stored at −20 °C until analysis. The NO_3_^−^-N concentrations were measured using a flow injection auto-analyzer (FLA star 5000 Analyzer, Foss, Denmark), after which PNA was calculated from the rate of linear regression of nitrate concentrations over time (mg NO_3_^−^-N g^−1^ h^−1^).

### Enzyme activity

The activities of 7 enzymes (acid phosphomonoesterase, sulfatase, β-glucosidase, β-cellobiosidase, N-acetyl-glucosaminidase, β-xylosidase, and α-glucosidase) were measured using 4-methylumbelliferyl-esters as substrates (Sigma-Aldrich, St. Louis, MO, USA), producing fluorescent 4-methylumbeliferone (MUF) after hydrolysis as described by Deng *et al*.^46^. Briefly, a soil suspension was prepared by adding 1 g of soil to 120 ml of deionized water. Aliquots (100 μl each) of the soil suspension were placed into microplate wells supplemented with 50 μl modified universal buffer at the pH optimal for each enzyme. Subsequently, 50 μl of 5 mM MUF-labeled substrate solutions were added to each microplate well. The well contents were well mixed before incubating at 37 °C for 1 h. The fluorescence intensity was quantified using a microplate fluorometer (Scientific Fluoroskan Ascent FL, Thermo) with 365 nm excitation and 450 nm emission filters. Enzyme activities were expressed as nanomoles h^−1^ g^−1^.

### Sequence processing

Sequences were processed using the initial process of RDP pipeline to remove low quality (Q score <20) and short reads (<300 bp)^47^. Chimera reads were removed through Uchime^48^ running in de novo mode. Sequences were filtered using FrameBot to remove non-*amoA* sequences^49^. Sequences were clustered based on CD-HIT using operational taxonomic units (OTU) pick up program in QIIME with nucleotide sequence dissimilarity at 20%^50^,^51^. Singletons were filtered and all samples were rarefied to the same number of reads as the sample with the lowest reads (1684 AOA and 2979 AOB reads per sample). Singletons were defined where only one sequence was present among all samples. All sequences were deposited in the NCBI Sequence Read Archive (SRA) database (Accession number: SRR1950559 and SRR1950564).

### Data analysis

Detrended correspondence analysis (DCA) was performed to examine beta-diversities (Bray-Curtis distances) between individual samples and for correlations between environmental factors including soil enzyme activities and community variation. Permutational multivariate analyses of variance (PERMANOVA)^52^ was performed to determine the significance of community composition differences between treatments. All AOA and AOB OTUs were illustrated using a heatmap. A phylogenetic tree was constructed based on the representative sequences of all the OTUs and *amoA* sequences downloaded from NCBI, based on MEGA^53^. Statistical analysis was performed using IMB SPSS statistics Version 20 (IBM Corporation, New York, United States), and complementary calculations were carried out in Microsoft Excel 2003. For each variable measured in the soil, the data were analyzed by one-way ANOVA using Tukey’s HSD test (p < 0.05).

## Additional Information

**How to cite this article**: Xue, C. *et al*. Quantitative and compositional responses of ammonia-oxidizing archaea and bacteria to long-term field fertilization. *Sci. Rep.* **6**, 28981; doi: 10.1038/srep28981 (2016).

## Figures and Tables

**Table 1:** Soil characteristics of control (CK), chemical fertilizer (NPK), and NPK plus organic fertilizer (NPKM) treatments.

**Soil characteristics**	**CK**	**NPK**	**NPKM**
pH	7.53 ± 0.15a	5.96 ± 0.09b	7.59 ± 0.07a
TN (%)	0.14 ± 0.01b	0.14 ± NAb	0.25 ± NAa
SOM (mg/g)	24.64 ± 0.37b	22.78 ± 0.47c	42.35 ± 0.33a
C/N	10.2 ± 0.34a	9.51 ± 0.22b	9.84 ± 0.05ab
AP (mg/Kg)	5.05 ± 0.05c	6.30 ± 0.08b	6.81 ± 0.09a
AK (mg/g)	0.17 ± NAa	0.26 ± 0.03a	0.34 ± 0.14a
PNA^*^ (mg NO_3_^−^ -N/kg dry soil h^−1^)	0.73 ± 0.21c	1.91 ± 0.16b	4.48 ± 0.76a

Numbers in the same column with the same letter are not significantly different (Tukey’s HSD, p > 0.05, n = 3). NA is less than 0.005.

^*^Potential nitrification activity.

**Table 2:** The activities (nmol h^−1^ g^−1^) of soil enzymes from control (CK), chemical fertilizer (NPK), and NPK plus organic fertilizer (NPKM) treatments.

**Treatment**	**CK**	**NPK**	**NPKM**
Acid phosphomonoesterase	141.75 ± 2.17b	202.64 ± 0.72a	106.38 ± 3.05c
*N*-acetyl-glucosaminidase	12.67 ± 3.48b	20.24 ± 2.38a	14.27 ± 1.13ab
Sulfatase	1.00 ± 0.04c	1.39 ± 0.08b	2.11 ± 0.10a
α-glucosidase	27.90 ± 0.96b	19.61 ± 0.68c	56.59 ± 3.10a
β-glucosidase	94.39 ± 14.66b	103.22 ± 1.60b	159.20 ± 7.95a
β-D-xylosidase	35.31 ± 3.75b	14.47 ± 1.31c	64.30 ± 1.55a
β-cellobiosidase	20.88 ± 2.16c	28.86 ± 1.66b	45.33 ± 3.02a

Numbers in the same column with the same letter are not significantly different (Tukey’s HSD, p > 0.05, n = 3).
